# Investigation of Surfactant-Polymer Interactions Using Rheology and Surface Tension Measurements

**DOI:** 10.3390/polym12102302

**Published:** 2020-10-08

**Authors:** Jia Yang, Rajinder Pal

**Affiliations:** Department of Chemical Engineering, University of Waterloo, Waterloo, ON N2L 3G1, Canada; j272yang@uwaterloo.ca

**Keywords:** polymer, surfactant, surfactant-polymer interaction, rheology, shear-thinning, non-Newtonian, viscosity, surface tension

## Abstract

The interactions between surfactants and a drag-reducing polymer were investigated at a low polymer concentration of 500 ppm, using measurements of the rheology and surface activity of surfactant-polymer solutions. A well-known drag-reducing polymer (anionic sodium carboxymethyl cellulose) and five different surfactants (two anionic, two non-ionic, and one zwitterionic) were selected for the interaction studies. The surfactant-polymer solutions were shear thinning in nature, and they followed the power law model. The interaction between the surfactant and polymer had a strong effect on the consistency index of the solution and a marginal effect on the flow behavior index. The surface tension versus surfactant concentration plots were interpreted in terms of the interactions between surfactant and polymer. The critical aggregation concentration (CAC) of the surfactant was estimated based on the surface tension and rheological data. The CAC values of the same charge surfactants as that of the polymer were found to be significantly higher than other combinations of surfactant and polymer, such as non-ionic surfactant/anionic polymer, and zwitterionic surfactant/anionic polymer.

## 1. Introduction

Mixtures of polymers and surfactants are used in a variety of applications, such as drug delivery [1,2,3], oil recovery [4], cosmetics, and more [5,6]. In general, the mixtures of polymers and surfactants are used to control the rheology of the solution, and to manipulate surface adsorption of the surfactant. The presence of a polymer in a solution may help to remove a surfactant from a surface, or improve its adsorption at the surface. In many applications, a suitable rheology, such as thickening of the solution or gelation of the solution, is required. The combination of surfactant and polymer allows easy manipulation and control of the solution rheology. The interaction of surfactant molecules with the polymer macromolecules can increase or decrease the solution viscosity due to stretching or shrinking of polymer chains. The presence of polymer can also speed up the micellization process, resulting in a decrease in the free surfactant concentration. This property is often exploited in skin formulations, as any free surfactant molecules tend to harm the skin and cause irritation. 

Mixtures of polymer and surfactant are also exploited to enhance frictional drag reduction in the turbulent flow of liquids [7,8,9]. When a small quantity (in ppm) of soluble polymer (such as sodium carboxymethyl cellulose) is added to a pipeline turbulent flow of liquids (such as water), a significant reduction in friction, and hence pumping costs, is observed. This phenomenon of drag reduction (DR) by polymeric additives has been utilized in the pipeline flow of liquids, wastewater treatment, sludge technology, heating and cooling loops, hydraulic and jet machinery, biomedical applications, and more [10,11,12,13,14]. The DR effect exhibited by polymers is explained in different ways. According to some researchers [15,16,17], the addition of polymer to a liquid increases the elongational viscosity of the liquid, due to the resistance to stretching offered by the polymer chains. The increase in elongational viscosity leads to an increase in the thickness of the buffer layer in turbulent flow, resulting in a reduction of wall friction. According to some authors [18], polymer chains cause suppression of turbulence, and consequently a decrease in turbulent drag reduction. The energy of turbulent eddies (velocity fluctuations) is taken up by the polymer chains in the form of stretching of the polymer chains. The stretched chains dissipate the stored elastic energy into heat upon relaxation to equilibrium state. While drag reduction in a turbulent flow of liquids due to soluble polymers is well established, only a handful of studies have been published on the synergistic effects of mixed surfactant-polymer systems. Pal’s group [7,8,9] published several articles recently to demonstrate that the addition of surfactant to polymer solution gives a significantly higher drag reduction effect when compared with polymer alone. When anionic surfactant sodium dodecyl sulfate (SDS) is added to non-ionic polymer polyethylene oxide (PEO), a percentage reduction in friction factor as high as 79 can be achieved [8]. When cationic surfactant octadecyltrimethylammonium chloride (OTAC) is added to non-ionic PEO, the drag reduction is enhanced [7]. One commonly encountered problem with polymeric drag reducing additives is that they rapidly undergo mechanical degradation in a turbulent flow field, and hence become ineffective as drag reducers. Mohsenipour and Pal [9] have shown that the addition of surfactant to polymer solution not only intensifies the drag reduction effect, it also increases the resistance of polymer molecules against mechanical (shear) degradation. According to Mohsenipour and Pal [9], the coiled polymer molecules undergo faster mechanical degradation compared with stretched polymer molecules. Thus, the study of surfactant-polymer interactions is important in the formulation of effective drag reducing additives. 

Surfactants consist of both hydrophilic (head) and hydrophobic (tail) groups. They can be classified into four groups, depending on the charge of their head group: anionic, cationic, non-ionic, and zwitterionic. If there is no charge group on its head, the surfactant is a non-ionic surfactant. An ionic surfactant carries a net charge on the head group. If the charge is only of one kind (positive or negative), the surfactant is called an anionic, or cationic, surfactant. If a surfactant contains a head with two oppositely charged groups, it is termed a zwitterionic surfactant [19]. Surfactants are used extensively in many industries such as food, cosmetics, detergents, paint, pharmaceuticals, petroleum, and more. A rapid growth in the usage of surfactants is anticipated in the years to come. More recently, surfactants have been used for environmental remediation, for the removal of various contaminants from polluted soils and aquifer sediments [20,21,22,23,24]. Surfactants have also been found to be effective drag reducers. Like polymers, surfactants can reduce frictional drag in a turbulent flow of liquids. However, drag reduction by surfactants has not received as much attention as polymers, by comparison.

Polymer-surfactant interaction studies, dealing with polymer-surfactant aggregation, are usually carried out at low surfactant concentrations [25,26,27,28]. The interaction of polymer and surfactant depends on the types of polymer and surfactant, and the solution conditions [29,30]. The interactions between polymer and surfactant can be broadly categorized into two groups: (a) electrostatic interactions, and (b) hydrophobic interactions. The interactions between ionic polymers and oppositely charged ionic surfactants are electrostatic in nature. The interactions between non-ionic polymer and ionic/non-ionic surfactants are hydrophobic in nature [25,31,32]. In hydrophobic interactions, the interaction occurs between the hydrophobic parts of the polymer and surfactant molecules.

When a surfactant is present in a polymer solution, the interaction of polymer and surfactant molecules begins at a certain surfactant concentration called the “critical aggregation concentration” (CAC). The CAC is usually lower than the critical micelle concentration (cmc) of the surfactant solution alone [25,31,32,33]. The CAC is significantly lower than the cmc of the surfactant when electrostatic interactions between an ionic polymer and an oppositely charged ionic surfactant are involved. The CAC is close to the cmc in the case of hydrophobic interactions between non-ionic polymers and ionic/non-ionic surfactants. While CAC reflects the onset of the interaction between polymer and surfactant, there is another critical surfactant concentration, referred to as the polymer saturation point (PSP), where the polymer chains become saturated with bound surfactant molecules or micelles [19,31]. When the interactions between surfactant and polymer are weak, CAC and PSP values are usually close to the cmc of the pure surfactant [19]. The critical concentrations (cmc, CAC, PSP) are often determined by surface tension and electrical conductivity measurements. The plots of surface tension and electrical conductivity show different break points as the surfactant concentration of the polymer solution is increased [19]. 

When surfactant is introduced to a polymer solution, interactions between the surfactant and the polymer lead to the formation of surfactant-polymer aggregates. Nagarajan [34] suggested a “necklace model” to explain the aggregations between polymer and surfactant micelles. According to the “necklace model”, the polymer macromolecule warps around the surfactant micelles. The polymer segments penetrate into the polar head group of micelles, and protect the hydrophobic tails from contacting water. The surfactant-polymer interactions can lead to the extension of polymer macromolecules. The surfactant molecules interact with the polymer macromolecule at favorable sites, form micelles, and open up and extend the coiled macromolecule. Thus, surfactant-polymer interactions can have a strong influence on the rheological behavior of solutions, due to extension, shrinking, and bridging of polymer macromolecules. Although a number of studies have been published on surfactant-polymer interactions, the complex behavior of mixed additives in solutions is far from being well understood. 

The broad objective of this work was to explore the interactions between different surfactants and a polymer, with frictional drag reduction in turbulent flows as the final application. To that end, a well-known drag reducing polymer, anionic sodium carboxymethyl cellulose, was selected for the interaction studies. Sodium carboxymethyl cellulose has received widespread attention in the literature due to its excellent drag reduction performance and shear resistance [35]. The interactions of the selected polymer with five different surfactants (two anionic, two non-ionic, and one zwitterionic) were investigated through studies of the rheology and surface activity of surfactant-polymer solutions. To the best of our knowledge, the interactions between sodium carboxymethyl cellulose and the surfactants selected in this work have not been reported before in the literature. As drag reduction by polymeric additives usually occurs at very low concentrations of polymer (of the order of ppms), the polymer concentration was fixed at a low value of 500 ppm (0.05 wt%) in the surfactant-polymer interaction experiments. At this low concentration of polymer, that is 0.05 wt%, the only rheological property that can be measured accurately is the shear viscosity. Any other rheological property (for example, viscoelastic properties, such as storage modulus or normal stresses), if at all possessed by such a dilute polymer solution, was expected to be too small to be detected accurately in a standard rheometer. Thus, the rheological measurements of polymer and surfactant-polymer solutions carried out in this work were restricted to shear viscosity measurements over a range of shear rates. 

## 2. Experimental Work

### 2.1. Materials

**Figure 1:**
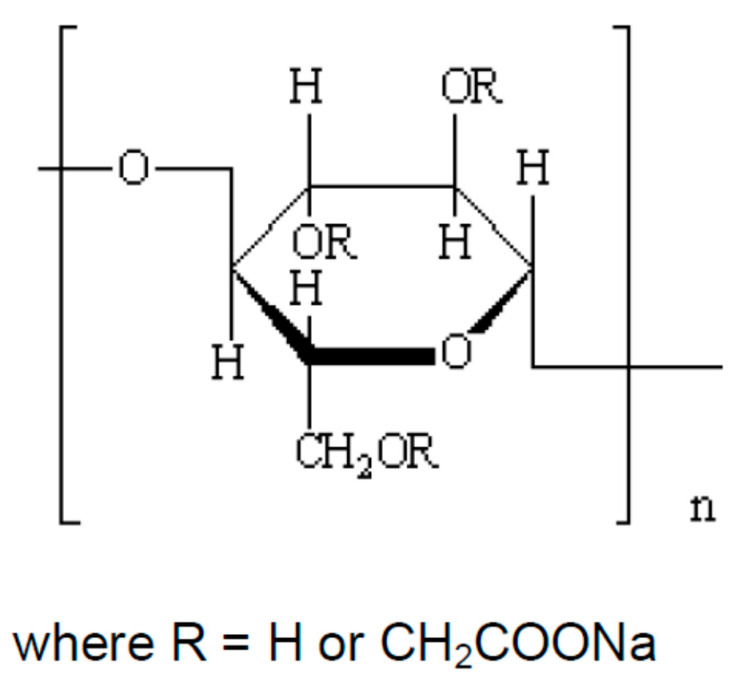
The structure of the repeating unit of Aqualon CMC (CMC) where R=H or ${\mathrm{CH}}_{2}\mathrm{COONa}$.

The polymer used in this work was Aqualon CMC (referred to as simply CMC). Aqualon CMC is purified sodium carboxymethyl cellulose. The chemical structure of CMC is shown in Figure 1. CMC is an anionic water soluble polymer, used extensively in applications related to food, pharmaceuticals, cosmetics, personal care, paper, and many more industries. It is often used as a rheology modifier or thickener to improve the stability of suspension and emulsion type products. It is manufactured by Ashland Inc., Covington, KY, USA. CMC is produced by reacting alkali cellulose with sodium monochloroacetate under strictly controlled conditions. It has a minimum purity of 99.5%. The molecular weight of CMC is approximately 700,000, and the degree of substitution is 0.7.

Five different surfactants were investigated for the interaction experiments. They are described in Table 1. Deionized water was used as a solvent for the polymers.

### 2.2. Procedures

Solutions of pure polymer (CMC) and mixtures of surfactant and polymer were prepared at room temperature. For pure polymer solutions, the CMC concentration was varied from 100 to 2000 ppm. For solutions of mixtures of surfactant and polymer, the concentration of polymer was fixed at 500 ppm, but the concentration of surfactant was varied. All solutions were prepared using deionized water as the solvent. The additives were added slowly to the deionized water while maintaining gentle mixing until the additives were dissolved. The rheology and surface activity of each solution was studied.

A coaxial cylinder viscometer (Fann 35A/SR 12 viscometer, Fann Instrument Company, Houston, TX, USA) was used to obtain the shear stress versus shear rate data at room temperature (23 ± 2 °C). The inner cylinder (bob) of the viscometer was stationary and the outer cylinder (rotor) was rotated at a known speed (rpm). The rotor radius was 1.8415 cm and the bob radius was 1.7245 cm. The bob height was 3.8 cm and the shear gap, in which the fluid was sheared, was 0.117 cm. From the dial reading versus rpm data, the shear stress and shear rate were calculated. The shear stress versus dial reading relation (calibration) was obtained using the known viscosity standards. The shear rate at the surface of the bob was calculated from the following equation:

(1) $$\dot{\gamma }=\frac{2N}{1-S^{-2N}}\Omega$$

where $\dot{\gamma }$ is shear rate, N is the slope of ln(Ω) versus ln(τ) data, S is the ratio of rotor to bob radii (S=1.0678), τ is the shear stress, and Ω is the rotor speed in rad/s.

The surface tension measurements were carried out using a CSC Du Nouy ring tensiometer (model no 70535, CSC Scientific Company, Fairfax, VA, USA). To measure the surface tension, the Du Nouy ring was placed below the surface of the liquid, and the ring was pulled upward through the surface of the liquid. The force required to detach the ring from the surface of the liquid was measured precisely, and converted to surface tension. The surface tension of each solution was measured twice and the average value was calculated. The measurements were highly reproducible. The difference between the two measurements was negligible (<0.3 mN/m). The surface tension measurements of some liquid samples were also carried out using the ADSA (Axisymmetric Drop Shape Analysis) method. The values obtained by the Du Nouy ring method and the ADSA method were in close agreement with each other.

## 3. Results and Discussion

### 3.1. Rheological Behavior and Surface Activity of Solutions of Anionic Polymer CMC

**Figure 2:**
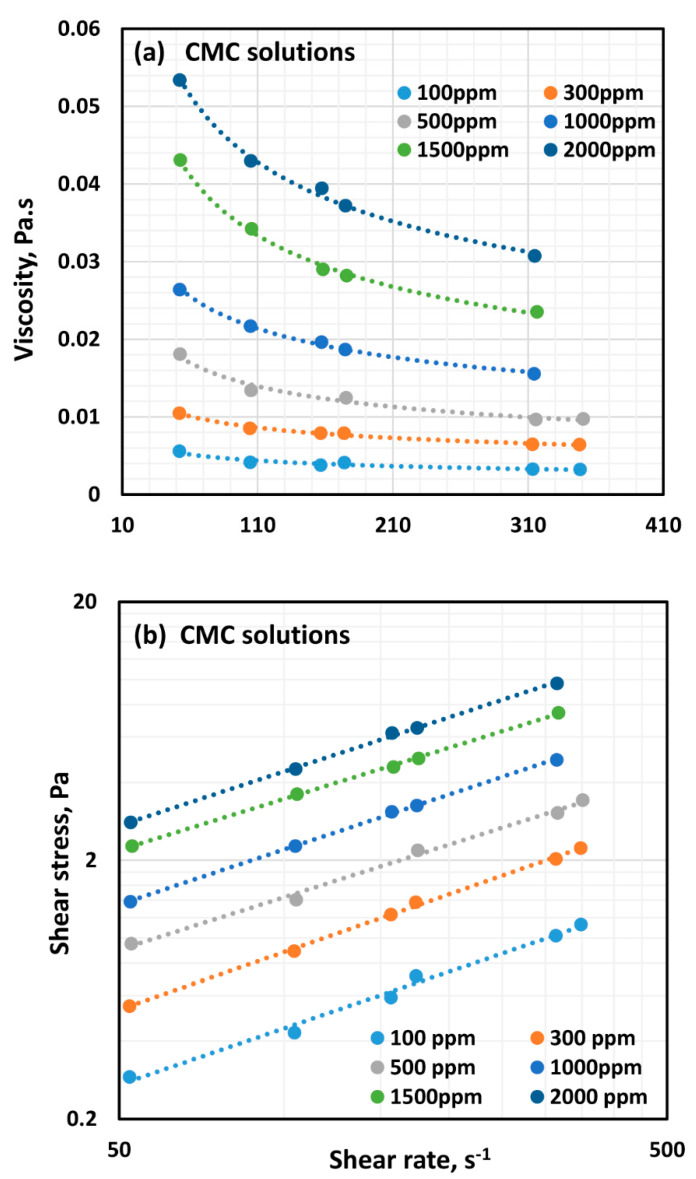
Flow curves for solutions of anionic polymer CMC at different polymer concentrations: (**a**) Viscosity versus shear rate and (**b**) Shear stress versus shear rate.

Figure 2 shows the viscosity versus shear rate (Figure 2a) and shear stress versus shear rate (Figure 2b) plots for solutions of anionic polymer CMC, for different values of polymer concentration. All polymer solutions are shear-thinning, in that the viscosity decreases with the increase in shear rate. The viscosity increases with the increase in polymer concentration at any given shear rate. Interestingly, the shear stress versus shear rate plots are straight lines on a log–log scale indicating the polymer solutions follow the power law behavior:

(2) $$\tau =K{\dot{\gamma }}^{n}$$

where τ is shear stress, $\dot{\gamma }$ is shear rate, K is consistency index, and n is flow behavior index. As the plots of shear stress versus shear rate are parallel, the flow behavior index, n, is nearly constant independent of the polymer concentration.

**Figure 3:**
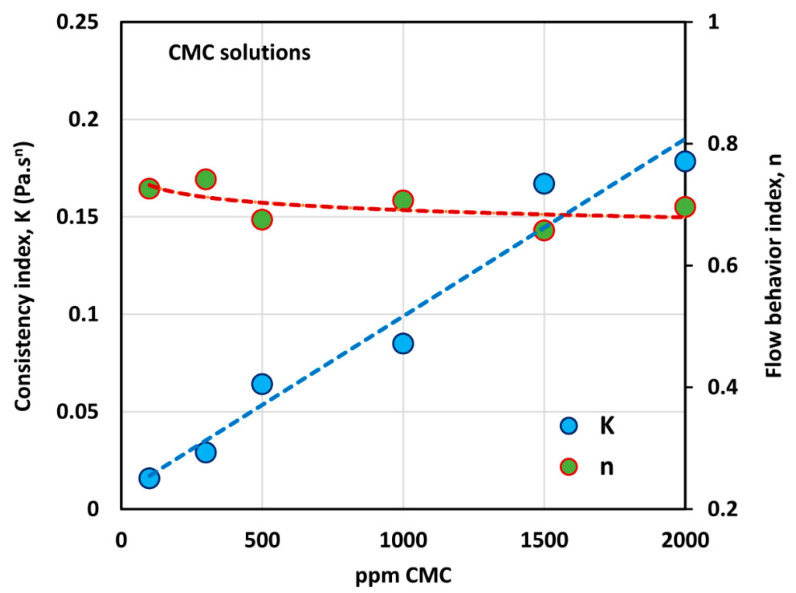
Variations of consistency index, K, and flow behavior index, n, with polymer (CMC) concentration.

Figure 3 shows the variations of consistency index, K, and flow behavior index, n, with the increase in polymer concentration. While the flow behavior index, n, decreases only marginally with the increase polymer concentration, the consistency index, K, shows a large increase with the increase in polymer concentration. The variation of K with the CMC concentration can be described by a linear relationship, as shown in the figure.

**Figure 4:**
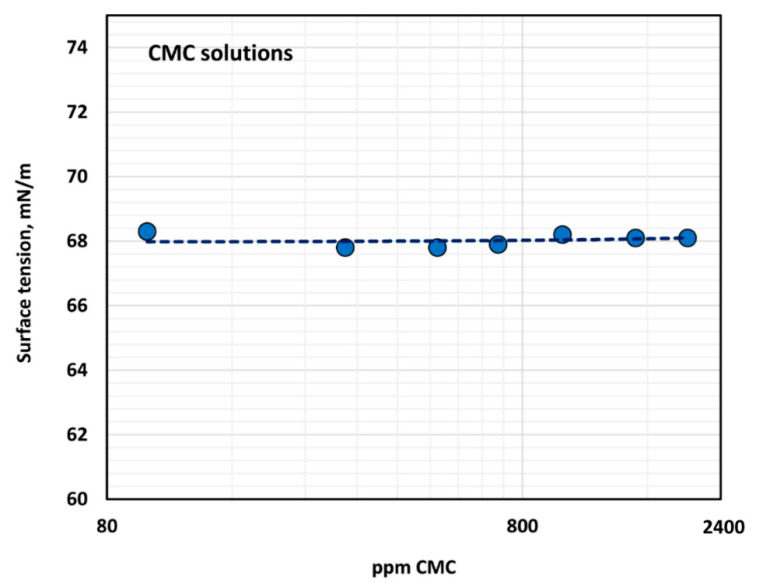
Variation of surface tension of polymer solution with the increase in polymer concentration.

The surface tension versus polymer concentration plot is shown in Figure 4. The CMC polymer solutions exhibit negligible surface activity as the surface tension is close to that of water, and there is no dependence of surface tension on the polymer concentration.

### 3.2. Effect of Anionic Surfactant (Stepwet DF-95) on the Rheological Behavior and Surface Activity of Solutions of Anionic Polymer CMC

**Figure 5:**
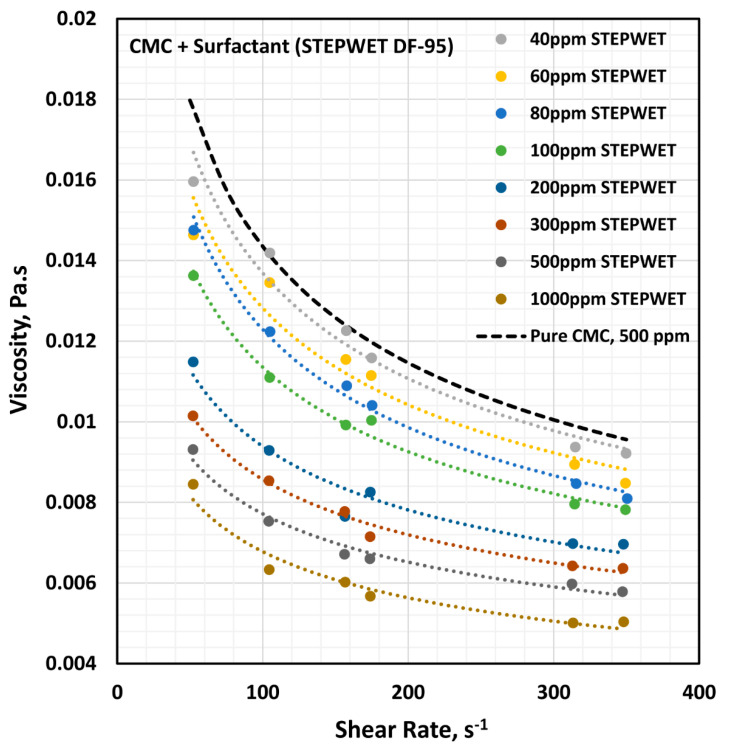
Effect of anionic surfactant (Stepwet DF-95) on the viscosity versus shear rate behavior of solutions of mixtures of the anionic polymer, CMC, and the anionic surfactant, Stepwet DF-95, at a fixed polymer concentration of 500 ppm.

**Figure 6:**
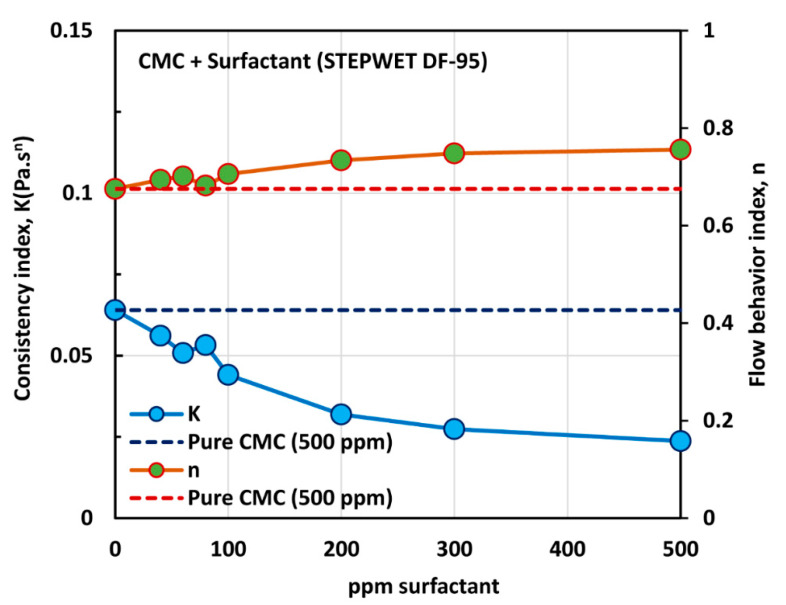
Variations of consistency index, K, and flow behavior index, n, for mixtures of anionic polymer, CMC, and anionic surfactant, Stepwet DF-95, with the increase in surfactant concentration, at a fixed polymer concentration of 500 ppm.

The influence of the anionic surfactant (Stepwet DF-95) on the viscosity of the anionic polymer (CMC) solutions at a fixed polymer concentration of 500 ppm is shown in Figure 5. The viscosity of the solution decreases with the increase in the surfactant concentration at any given shear rate. In addition, all solutions are shear-thinning. The flow curves (viscosity versus shear rate plots) at different surfactant concentrations are almost parallel to those of the pure polymer solution. The solutions followed the power law model (Equation (2)). The variations of consistency index, K, and flow behavior index, n, with the surfactant concentration are shown in Figure 6.

With the increase in the surfactant concentration, the flow behavior index, n, increases marginally, indicating that the solutions become less non-Newtonian. However, the consistency index, K, shows a large decrease with the increase in the surfactant concentration. Clearly the variations in K and n reflect a strong interaction between the surfactant molecules and polymer macromolecules. The presence of same charge (anionic) surfactant molecules in the solution does not provide a favorable environment for the anionic polymer chains to extend as much as in the absence of surfactant molecules. The repulsion between the same charge surfactant molecules and polymer chains causes shrinking of the polymer chains, resulting in a decrease in the consistency index, K, and an increase in the flow behavior index, n.

**Figure 7:**
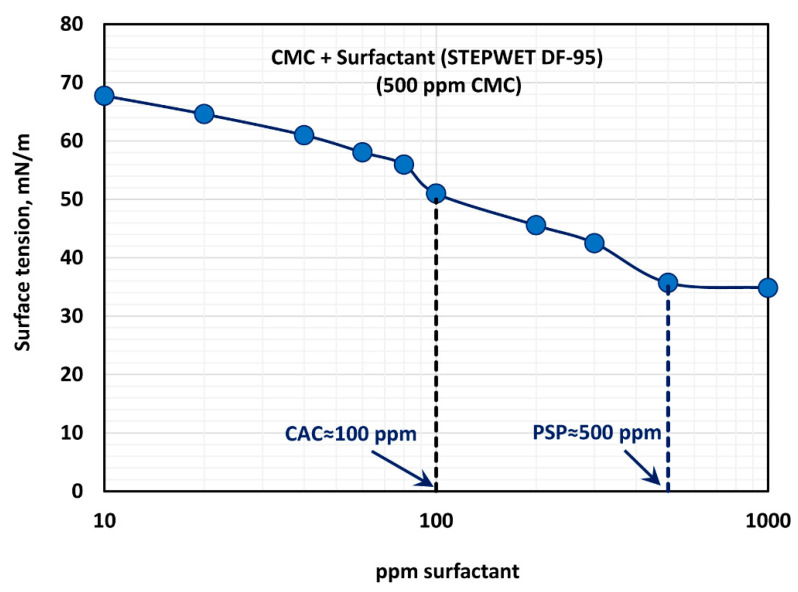
Variation of surface tension of surfactant-polymer solutions with the increase in surfactant concentration, at a fixed polymer concentration of 500 ppm.

The effect of surfactant concentration on the surface tension of surfactant-polymer solutions at a fixed polymer concentration of 500 ppm is shown in Figure 7. With the increase in surfactant concentration, the surface tension decreases rapidly and then levels-off at high surfactant concentration (> 500 ppm). The surface tension versus surfactant concentration plot shows two break points. The first break point, corresponding to CAC (critical aggregation concentration), occurs at a surfactant concentration of about 100 ppm, and the second break point corresponding to PSP (polymer saturation point concentration) occurs at a surfactant concentration of about 500 ppm. The surface tension behavior is consistent with the K and n plots of Figure 6. The consistency index, K, and the flow behavior index, n, undergo significant changes around 80–100 ppm surfactant concentration, and they level off around 500 ppm surfactant concentration.

### 3.3. Effect of Anionic Surfactant (Stepanol WA-100) on the Rheological Behavior and Surface Activity of Solutions of Anionic Polymer CMC

**Figure 8:**
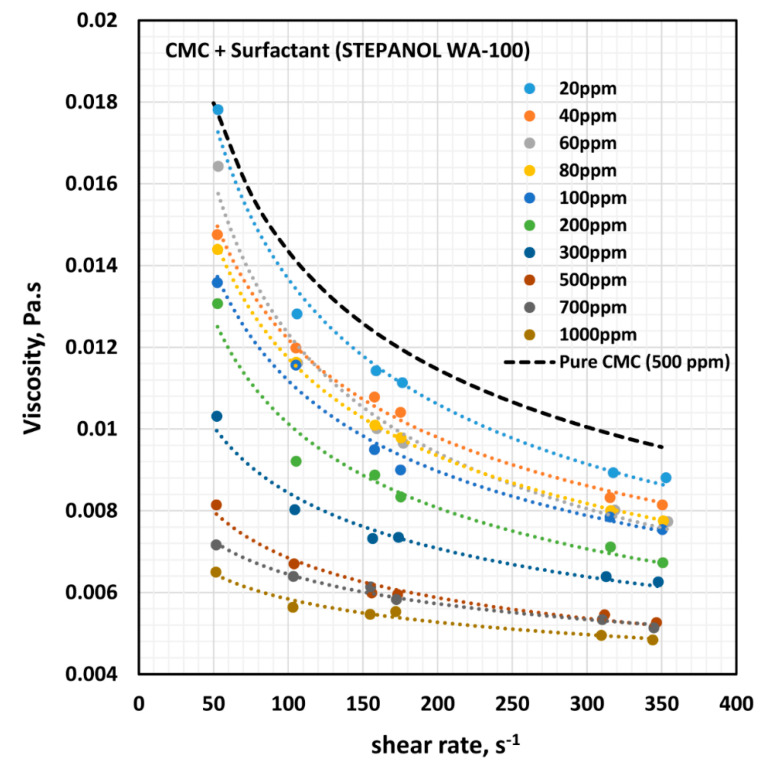
Effect of anionic surfactant (Stepanol WA-100) on the viscosity versus shear rate behavior of solutions of mixtures of anionic polymer, CMC, and anionic surfactant, Stepanol WA-100, at a fixed polymer concentration of 500 ppm.

**Figure 9:**
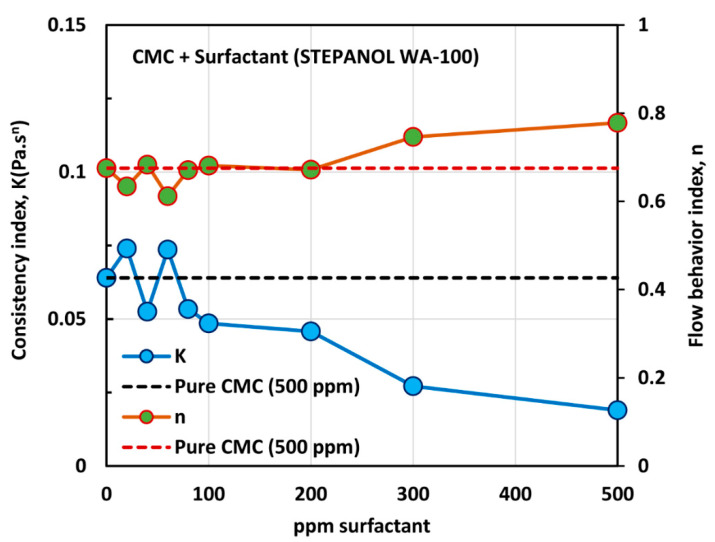
Variations of consistency index, K, and flow behavior index, n, for mixtures of anionic polymer CMC and anionic surfactant Stepanol WA-100 with the increase in surfactant concentration, at a fixed polymer concentration of 500 ppm.

Figure 8 shows the influence of anionic surfactant (Stepanol WA-100) on the viscosity of anionic polymer (CMC) solutions at a fixed polymer concentration of 500 ppm. The effect of Stepanol WA-100 on the viscosity of CMC polymer solution is similar to that of surfactant Stepwet DF-95. The solution viscosity decreases with the increase in the surfactant concentration and the solutions are shear-thinning. The solutions followed the power law model (Equation (2)). The variations of consistency index, K, and flow behavior index, n, with the increase in surfactant concentration are shown in Figure 9.

With the increase in the surfactant concentration, the flow behavior index, n, remains constant, equal to that of pure CMC solution, until the surfactant concentration of 200 ppm is reached. With further increase in surfactant concentration, the value of n increases, indicating that the solutions become less non-Newtonian. The consistency index, K, is nearly constant up to the surfactant concentration of about 80 ppm. With further increase in surfactant concentration, K undergoes a large decrease. The variations in K and n reflect a strong interaction between the surfactant molecules and polymer macromolecules at high surfactant concentrations. A similar behavior was observed in the case of Stepwet DF-95 surfactant. As explained earlier, the presence of the same charge (anionic) surfactant molecules in the solution does not provide a favorable environment for the anionic polymer chains to extend as much as in the absence of surfactant molecules. Consequently, the consistency index, K, decreases, and the fluid tends to become Newtonian.

**Figure 10:**
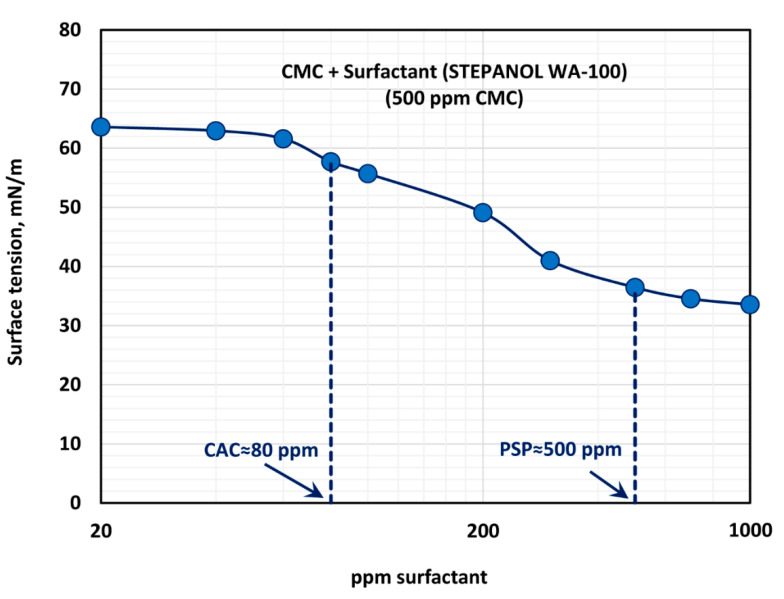
Variation of surface tension of surfactant-polymer solutions with the increase in surfactant (Stepanol WA-100) concentration, at a fixed polymer (CMC) concentration of 500 ppm.

Figure 10 shows the effect of surfactant (Stepanol WA-100) concentration on the surface tension behavior of surfactant-polymer solutions at a fixed polymer (CMC) concentration of 500 ppm. With the increase in surfactant concentration, the surface tension decreases rapidly and then levels-off at high surfactant concentration (> 500 ppm). From the surface tension versus surfactant concentration plot, it appears that the CAC is approximately 80 ppm where we observe the first break in surface tension-surfactant concentration plot. The PSP (polymer saturation point) where the surface tension tends to level off is 500 ppm. Note that these observations are consistent with the K and n plots shown in Figure 9. The values of K and n are nearly constant up to a surfactant concentration of about 80 ppm. At higher surfactant concentrations, K decreases and n increases until the surfactant concentration reaches 500 ppm.

### 3.4. Effect of Non-ionic Surfactant (Alfonic 1412-3 Ethoxylate) on the Rheological Behavior and Surface Activity of Solutions of Anionic Polymer CMC

**Figure 11:**
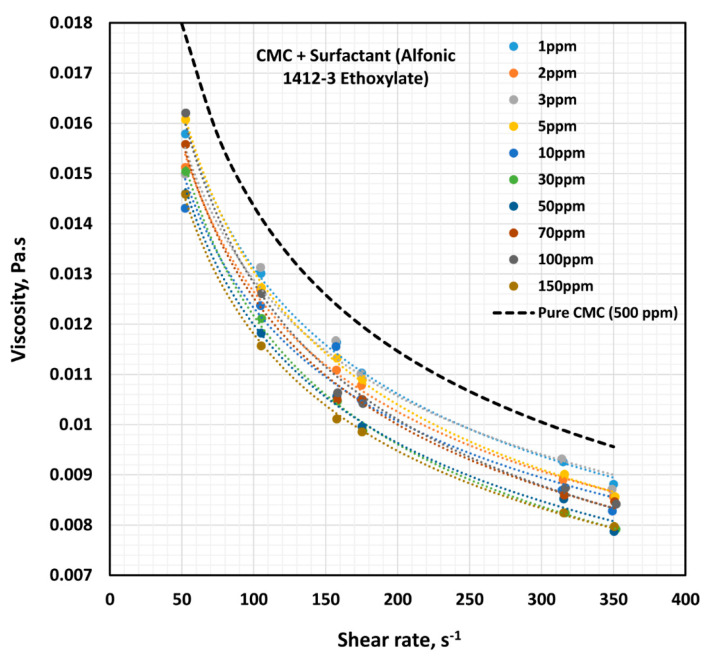
Effect of non-ionic surfactant (Alfonic 1412-3 Ethoxylate) on the viscosity versus shear rate behavior of solutions of mixtures of anionic polymer, CMC, and non-ionic surfactant, Alfonic 1412-3 Ethoxylate, at a fixed polymer concentration of 500 ppm.

**Figure 12:**
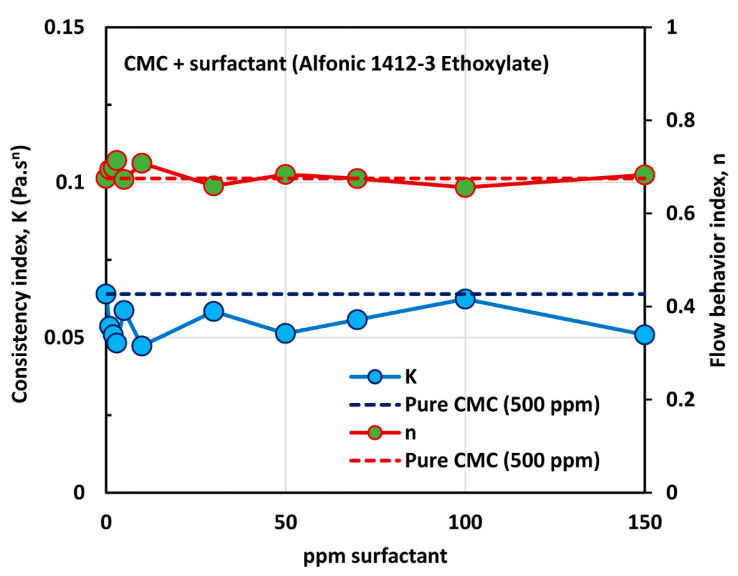
Variations of consistency index, K, and flow behavior index, n, for mixtures of anionic polymer, CMC, and non-ionic surfactant, Alfonic 1412-3 Ethoxylate, with the increase in surfactant concentration, at a fixed polymer concentration of 500 ppm.

The influence of non-ionic surfactant (Alfonic 1412-3 Ethoxylate) on the viscosity of anionic polymer (CMC) solutions at a fixed polymer concentration of 500 ppm is shown in Figure 11. All solutions are shear-thinning. The viscosity of the solution decreases with the increase in the surfactant concentration at any given shear rate. The flow curves (viscosity versus shear rate plots) at different surfactant concentrations are almost parallel to each other, indicating the same degree of shear thinning. Figure 12 shows the variations of consistency index, K, and flow behavior index, n. The flow behavior index, n, of the solution is not affected by the addition of surfactant. Note that the value of n for pure CMC solution at 500 ppm CMC is 0.675. However, the consistency index, K, drops by a significant amount upon the addition of surfactant to the polymer solution. Most of the drop in K occurs at low surfactant concentrations, of up to about 10 ppm. With further increase in surfactant concentration the value of K remains nearly constant. The decrease in the consistency index with the addition of surfactant is clearly indicative of surfactant-polymer interaction. It appears that the surfactant molecules aggregate on the polymer chains, and thereby reduce intra-repulsion between the same (negative) charges present on the polymer chain. The reduction of intra-repulsion causes shrinking of polymer chains, and hence a decrease in consistency.

**Figure 13:**
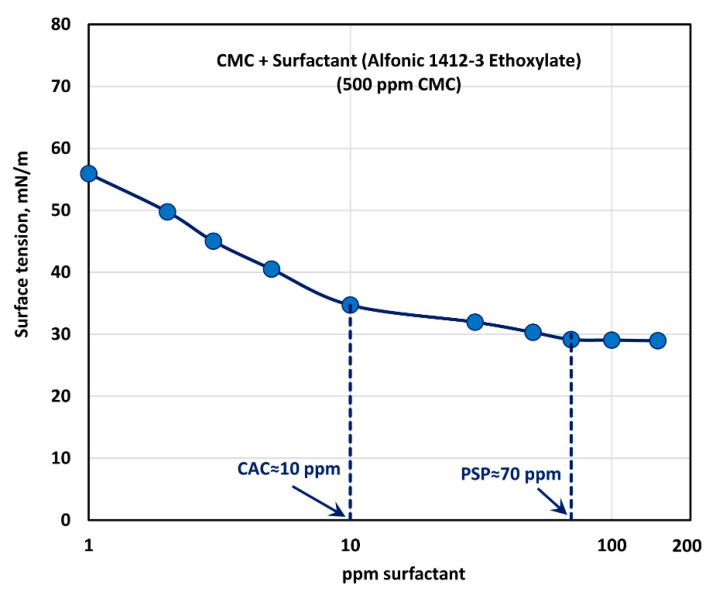
Variation of surface tension of surfactant-polymer solutions with the increase in surfactant (Alfonic 1412-3 Ethoxylate) concentration, at a fixed polymer (CMC) concentration of 500 ppm.

The effect of surfactant (Alfonic 1412-3 Ethoxylate) concentration on the surface tension behavior of surfactant-polymer solutions is shown in Figure 13 at a fixed polymer (CMC) concentration of 500 ppm. With the increase in the surfactant concentration, the surface tension decreases much more rapidly when compared with the other surfactants. Most of the drop in surface tension occurs within the surfactant concentration range of 0–10 ppm. Clearly the non-ionic surfactant, Alfonic 1412-3 Ethoxylate, is much more surface active compared with the other surfactants. The surface tension tends to level off when surfactant concentration exceeds 70 ppm. From the surface tension versus surfactant concentration plot, it appears that the CAC is approximately 10 ppm where we observe the first break in surface tension-surfactant concentration plot. The PSP (polymer saturation point) where the surface tension tends to level off is about 70 ppm.

### 3.5. Effect of Non-ionic Surfactant (Aromox DMC) on the Rheological Behavior and Surface Activity of Solutions of Anionic Polymer CMC

**Figure 14:**
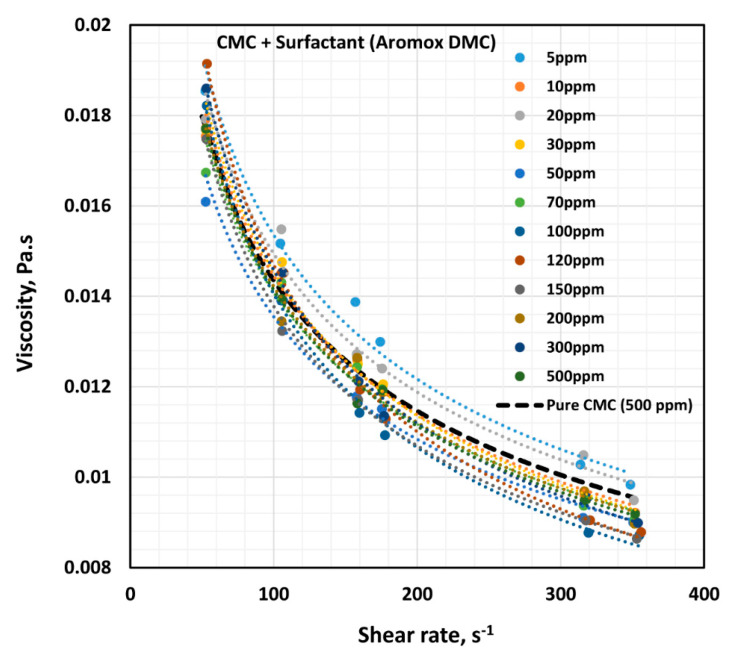
Effect of non-ionic surfactant (Aromox DMC) on the viscosity versus shear rate behavior of solutions of mixtures of the anionic polymer, CMC, and the non-ionic surfactant, Aromox DMC, at a fixed polymer concentration of 500 ppm.

**Figure 15:**
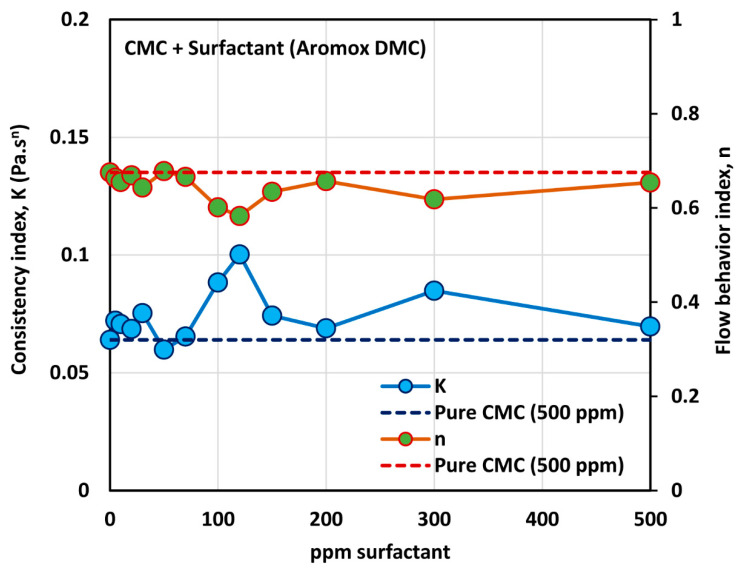
Variations of consistency index, K, and flow behavior index, n, for mixtures of anionic polymer, CMC, and non-ionic surfactant, Aromox DMC, with the increase in surfactant concentration, at a fixed polymer concentration of 500 ppm.

Figure 14 shows the influence of non-ionic surfactant Aromox DMC (Dimethylcocoalkylamine oxide) on the viscosity of anionic polymer (CMC) solutions at a fixed polymer concentration of 500 ppm. The solutions are shear thinning and the flow curves are nearly parallel to each other. Interestingly the flow curve for pure polymer solution falls below the flow curves of some of the surfactant-polymer solutions. The power law constants (K and n) are plotted in Figure 15. The flow behavior index, n, is nearly the same as that of the pure polymer solution until the surfactant concentration of about 80 ppm. With further increase in surfactant concentration, n falls below the pure polymer solution value. The consistency index, K, of surfactant-polymer mixture falls above the value of the pure polymer solution until a surfactant concentration of 30 ppm is reached. With increase in surfactant concentration from 50 to 120 ppm, the consistency index increases, and peaks at about 120 ppm. From these observations, it appears that the CAC (critical aggregation concentration) is well below 50 ppm, where the surfactant begins to interacts with the polymer molecules. The interaction of surfactant molecules with the polymer chains causes extension of the polymer chains, resulting in an increase in the value K. The PSP (polymer saturation point) is approximately 120 ppm, where a peak in the consistency index is observed.

**Figure 16:**
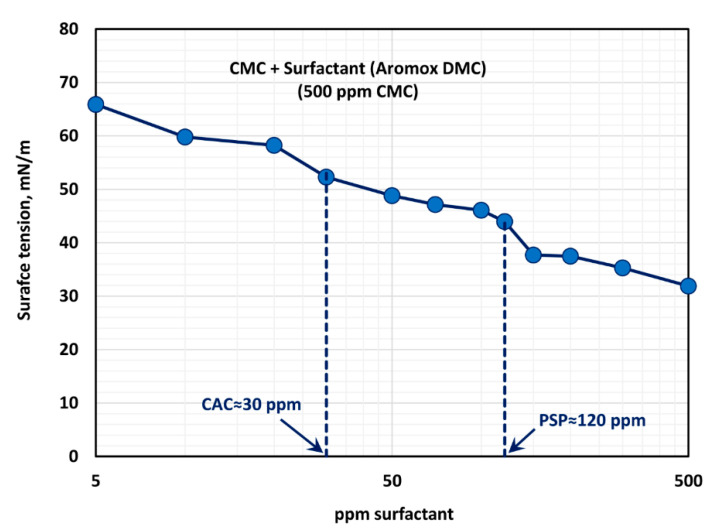
Variation of surface tension of surfactant-polymer solutions with the increase in surfactant (Aromox DMC) concentration, at a fixed polymer (CMC) concentration of 500 ppm.

The surface tension behavior of polymer (CMC) + surfactant (Aromox DMC) solutions is shown in Figure 16, at a fixed polymer (CMC) concentration of 500 ppm. With the increase in the surfactant concentration, the surface tension decreases as expected. Most of the drop in surface tension occurs within the surfactant concentration range of 0–150 ppm. The surface tension versus surfactant concentration plot shows a first plateau in the surfactant concentration range of approximately 30 to 120 ppm. From 120–150 ppm, the surface tension drops significantly and then it tends to level off (second plateau) at a higher surfactant concentration. Thus, the CAC and PSP values are approximately 30 ppm and 120 ppm, respectively. These observations are consistent with the consistency behavior of surfactant-polymer mixtures.

### 3.6. Effect of Zwitterionic Surfactant (Amphosol CG) on the Rheological Behavior and Surface Activity of Solutions of Anionic Polymer CMC

**Figure 17:**
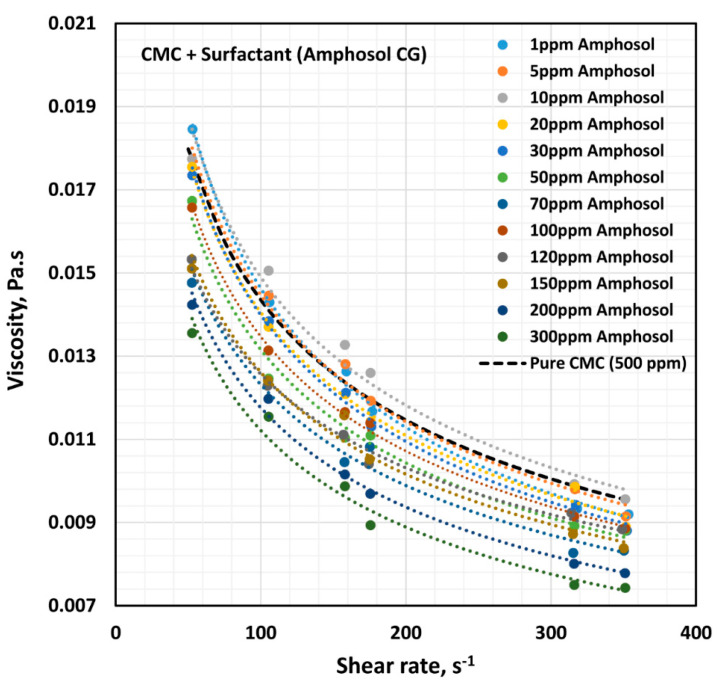
Effect of zwitterionic surfactant (Amphosol CG) on the viscosity versus shear rate behavior of solutions of mixtures of anionic polymer, CMC, and zwitterionic surfactant, Amphosol CG, at a fixed polymer concentration of 500 ppm.

**Figure 18:**
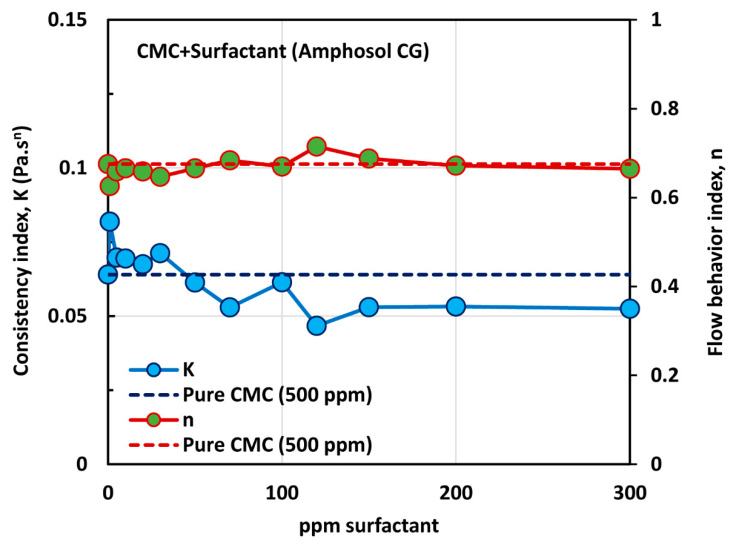
Variations of consistency index, K, and flow behavior index, n, for mixtures of anionic polymer, CMC, and zwitterionic surfactant, Amphosol CG, with the increase in surfactant concentration, at a fixed polymer concentration of 500 ppm.

The influence of zwitterionic surfactant (Amphosol CG) on the rheological behavior of surfactant-polymer solutions is shown in Figure 17. All solutions are shear thinning. The flow curve (viscosity versus shear rate) of the surfactant-polymer mixture solution generally falls below that of the pure polymer solution (CMC, 500 ppm), except at low concentrations of surfactant. The power-law constants (K and n) are plotted in Figure 18. The flow behavior index, n, is nearly the same as that of the pure polymer solution, throughout the surfactant concentration range. The consistency index, K, of the surfactant-polymer mixture is higher than that of the pure polymer solution at low surfactant concentrations of up to about 30 ppm. At higher surfactant concentrations, the consistency index, K, of the surfactant-polymer mixture falls significantly below that of the pure polymer solution. Thus, at low surfactant concentrations, the surfactant molecules cause extension of polymer chains, and at high surfactant concentrations, higher than 50 ppm, the interaction of surfactant molecules with the polymer chains results in shrinking of the polymer chains.

**Figure 19:**
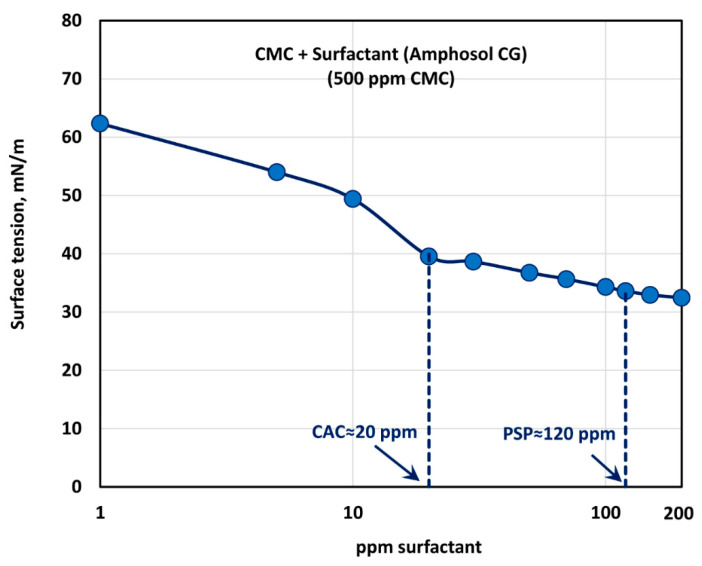
Variation of surface tension of surfactant-polymer solutions with the increase in surfactant (Amphosol CG) concentration, at a fixed polymer (CMC) concentration of 500 ppm.

Figure 19 shows the surface tension behavior of polymer (CMC) + surfactant (Amphosol CG) solutions at a fixed polymer (CMC) concentration of 500 ppm. With the increase in the surfactant concentration, the surface tension decreases rapidly up to a surfactant concentration of 20 ppm. With further increase in surfactant concentration, the surface tension decreases gradually up to a surfactant concentration of about 120 ppm, and then levels off. Thus, the critical aggregation concentration (CAC) is about 20 ppm, where the break in surface tension versus concentration plot is observed and the surfactant molecules begin to associate with the polymer chains. The polymer chains are saturated with the surfactant molecules at surfactant concentrations above 120 ppm. The consistency index, K, also becomes nearly constant above 120 ppm surfactant (see Figure 18).

### 3.7. Discussion

Table 2 summarizes the approximate CAC and PSP values of different surfactants, estimated from the surface tension versus surfactant concentration plots and rheological information. The critical micelle concentrations (cmc) of some of the pure surfactants are also shown. Interestingly, the CAC values for the same charge surfactant as polymer (that is, anionic surfactant/anionic polymer) are high (>80 ppm), as expected due to repulsion between surfactant and polymer molecules. Likewise, the PSP concentrations are high (500 ppm) for the same charge surfactants as polymer. For non-ionic and zwitterionic surfactants, the CAC values are small in the range of 10–30 ppm. It should also be noted that CAC values are significantly lower than the cmc of pure surfactants whose cmc values are known.

The interactions between CMC and different surfactants are summarized in Table 3, where the symbol “S” refers to surfactant, “P” refers to polymer, the superscripts refer to the charge on the species (0 for neutral, − for negative charge, and + for positive charge).

## 4. Conclusions

The interactions between the polymer and the surfactants were explored experimentally using rheology and surface tension measurements. The polymer studied was anionic sodium carboxymethyl cellulose (CMC). The surfactants studied were: anionic Stepwet DF-95, anionic Stepanol WA-100, Non-ionic Alfonic 1412-3 Ethoxylate, Non-ionic Aromox DMC, and Zwitterionic Amphosol CG. Based on the experimental work, the following conclusions can be made:

- The interactions between anionic surfactants and an anionic polymer are strong in terms of the consistency index. The consistency index decreases with the addition of surfactant to the polymer. However, the CAC values of anionic surfactants are significantly higher than the other combinations of surfactant and polymer investigated.
- The interactions between the following combinations of surfactant and polymer are weak in terms of the consistency index: non-ionic surfactant Alfonic 1412-3 Ethoxylate/anionic polymer CMC, and zwitterionic surfactant Amphosol CG/anionic CMC. The consistency index generally decreases with the addition of surfactant to polymer.
- The interactions between the non-ionic surfactant, Aromox DMC, and the anionic polymer, CMC, are moderate in terms of the consistency index. However, in this case, the consistency index increases with the addition of surfactant to the polymer, indicating an increase in the hydrodynamic size of the polymer molecules. Thus, this combination of surfactant and polymer is promising from a drag reduction point of view.
- The CAC values of anionic surfactants and anionic polymer are in the range of 80–100 ppm.
- The CAC values of other combinations of surfactants (non-ionic, zwitterionic) and anionic polymer are in the range of 10–30 ppm.
- For the surfactants of known critical micelle concentration (cmc), the CAC values were found to be significantly lower than the cmc.

## Figures and Tables

**Table 1:** Different surfactants investigated in this work.

Trade Name	Chemical Name or Structure	Type of Surfactant and Additional Information	Industrial Uses and Manufacturer
Alfonic 1412-3 Ethoxylate	Ethoxylated alcohol, CH_3_(CH_2_)_x_CH_2_(OCH_2_CH_2_)_3_OH where ‘x’ varies between 10 and 12	Non-ionic, liquid, no salt present, 100% active, critical micelle concentration (cmc) of 48.2 mg/L, surface tension of 21.7 mN/m above cmc.	Used in liquid detergents, hard surface cleaners, and other industrial cleaning formulations. Additionally used as a wetting agent, emulsifier, and degreaser. It is manufactured by Sasol Chemicals, Houston, TX, USA
Aromox DMC	Dimethylcocoalkylamine oxide	Non-ionic, liquid, Amine oxide −38 to 43%, Amine−1.5% max, peroxide 0.34% max, surface tension of 30 mN/m above cmc.	Used as a thickener. It is manufactured by AkzoNobel, Amsterdam, Netherlands
Stepwet DF-95	Sodium Lauryl Sulfate based surfactant; CH_3_ (CH_2_)_10_ CH_2_ OSO_3_ Na	Anionic, solid powder, minimum 93% active, sodium lauryl sulfate >93%, sodium sulfate <3.5%, sodium chloride < 2%	Used as a wetting agent. It is manufactured by Stepan Company, Northfield, IL, USA
Stepanol WA-100	Sodium Lauryl Sulfate based surfactant; CH_3_ (CH_2_)_10_ CH_2_ OSO_3_ Na	Anionic, solid powder, sodium lauryl sulfate 97–100%, sodium sulfate 0.56%, sodium chloride 0.025%, 97.59% active, cmc of 389 mg/L, surface tension of 36.3 mN/m above cmc.	Used as a foaming agent and mouth dispersant in dentifrices. Used in powdered formulations, such as hand cleaners and powdered baths. Can be used in liquid hand soaps and shampoos. It is manufactured by Stepan Company, Northfield, IL, USA
Amphosol CG	Cocamidopropyl Betaine (CAPB); CAPB is a fatty acid amide consisting of a long hydrocarbon chain at one end and a polar group at the other.	Zwitterionic consisting of both quaternary ammonium cation and a carboxylate, aqueous liquid, 30% active (30% CAPB), surface tension of 29.3 mN/m above cmc.	Used as a humectant, foam booster, antistatic agent, viscosity builder. Used in bubble baths, hand soaps, hair conditioners, cleansing creams and lotions, cream rinses, shower gels, shampoos. It is manufactured by Stepan Company, Northfield, IL, USA

**Table 2:** Summary of approximate values of CAC and PSP for different surfactants.

Surfactant	Polymer	CAC in ppm	PSP in ppm	Surfactant Critical Micelle Concentration (cmc)
Anionic (Stepwet DF-95)	Anionic CMC	100	500	Not available
Anionic (Stepanol WA-100)	Anionic CMC	80	500	390 mg/L ≈ 390 ppm
Non-ionic (Alfonic 1412-3 Ethoxylate)	Anionic CMC	10	70	48.2 mg/L ≈ 48.2 ppm
Non-ionic (Aromox DMC)	Anionic CMC	30	120	Not available
Zwitterionic (Amphosol CG)	Anionic CMC	20	120	Not available

**Table 3:** Summary of interactions between polymer and surfactants.

Surfactant	Polymer	Surfactant -Polymer Combination	Comments
Anionic (Stepwet DF-95)	Anionic (CMC)	**S^−^ P^−^**	Strong interaction observed between surfactant and polymer based on consistency index; consistency decreases upon addition of surfactant
Anionic (Stepanol WA-100)	Anionic (CMC)	**S^−^ P^−^**	Strong interaction observed between surfactant and polymer based on consistency index; consistency decreases upon addition of surfactant
Non-ionic (Alfonic 1412-3 Ethoxylate)	Anionic (CMC)	**S^0^ P^−^**	Weak interaction observed between surfactant and based on consistency index; consistency decreases upon addition of surfactant
Non-ionic (Aromox DMC)	Anionic (CMC)	**S^0^ P^−^**	Moderate interaction observed between surfactant and polymer based on consistency index; consistency increases upon addition of surfactant
Zwitterionic (Amphosol CG)	Anionic (CMC)	**S^+-^ P^−^**	Weak interaction observed between surfactant and polymer based on consistency index; consistency decreases upon addition of surfactant
